# Frederick John Poynton

**Published:** 1944

**Authors:** 


### FREDERICK JOHN POYNTON, M.D. (Lond.), F.R.C.P.

FREDERICK JOHN POYNTON was born at Kelston rectory, Bath, in 1869. On leaving Marlborough College he started his medical education at University College, Bristol, and continued it at St. Mary's Hospital where he won a scholarship in Anatomy and Physiology. He qualified in 1893, and rapidly showed the absorbing interest in children which largely determined his medical career. A disciple of Cheadle and Barlow, the two great figures in British Pædiatrics at the end of the last century, he was appointed to the staff of Great Ormond Street in 1900, and in 1903 became assistant physician to University College Hospital with charge of the Children's ward. He loved children and children loved him. In 1931, when President of the British Pædiatric Association, he said that "he who has brought a smile to the face of a dying child has had a glimpse of the eternal." Those who have been privileged to see Poynton handling a sick child will know that he must have had many such glimpses.

Poynton enjoyed teaching and his enthusiasm, frankness of speech and essential honesty of purpose endeared him to all students. Many of his old house physicians and registrars owe much of their later success in life to his tremendous inspiration and encouragement. The famous Saturday morning rounds at Great Ormond Street will never be forgotten by those who participated in them and, judging from the number of overseas visitors who attended, they must at one time have had an almost international fame.

His great work on the ætiology of acute rheumatism perhaps reached its climax in 1913 when he published in *Researches on Rheumatism* the collected results of fifteen years' work. His main thesis was that acute rheumatism is due to a streptococcus. This view has, of course, been seriously attacked, but recent work has gone far to show that some such organism probably does play an important part in the production of the disease. Poynton never maintained, as his critics allege, that this organism was the only factor concerned. The acute controversy over this question has now largely died down and only time will show whether his view is correct or not. However, the most important and valuable part of his work was undoubtedly the interest and attention which he focussed on acute rheumatism. The realization of the importance of this disease in the causation of heart disease and our schemes for hospital schools and supervisory clinics are largely due to the work and enthusiasm of Poynton and his disciples.

His service to pædiatrics was recognized in 1930 by the award of the Dawson Williams Prize of the British Medical Association for *Advances in the Study of Children's Diseases in the British Empire.* He gave the Bradshaw Lecture at the Royal College of Physicians in 1924, and was Senior Censor in 1930. He was Lettsomian Lecturer in 1927, and Harben Lecturer in 1936.

Apart from his medical success Poynton achieved fame in quite another sphere. He played cricket for Somerset from 1891 to 1896 and hockey for Middlesex in 1894.

Poynton had associations with Bristol in addition to starting his medical studies here. In 1934 he gave the Long Fox Lecture on "Heart Disease in Childhood." He was consulting physician to Winford Orthopædic Hospital and always took a keen interest in the progress of the hospital. In the early days of his retirement to Bath he often motored over to see the cardiac patients.

Poynton, despite his long sojourn in London remained at heart a country man and was firmly attached to the West country. When the time came for him to retire from active practice he returned to the haunts of his childhood and settled in Bath. Here, after a narrow escape in an air-raid, he died of a heart attack on October 29th, 1943. His clinical enthusiasm and keenness persisted right to the end and only a few weeks before his death he published a stimulating and unorthodox paper on joint changes in acute anterior polio-myelitis, the result of observations made at the Bath and Wessex Orthopædic Hospital.

With his death we have lost one of the great men in medicine of the century.

